# Shear Stress Affects Biofilm Structure and Consequently Current Generation of Bioanode in Microbial Electrochemical Systems (MESs)

**DOI:** 10.3389/fmicb.2019.00398

**Published:** 2019-03-06

**Authors:** Jiawei Yang, Shaoan Cheng, Chaochao Li, Yi Sun, Haobin Huang

**Affiliations:** State Key Laboratory of Clean Energy, Department of Energy Engineering, Zhejiang University, Hangzhou, China

**Keywords:** anode biofilm, shear stress, anode performance, viability, microbial community

## Abstract

Shear stress is an important factor that affects the formation and structure of anode biofilms, which are strongly related to the extracellular electron transfer phenomena and bioelectric performance of bioanodes. Here, we show that using nitrogen sparging to induce shear stress during anode biofilm formation increases the linear sweep voltammetry peak current density of the mature anode biofilm from 2.37 ± 0.15 to 4.05 ± 0.25 A/m^2^. Electrochemical impedance spectroscopy results revealed that the shear-stress-enriched anode biofilm had a low charge transfer resistance of 46.34 Ω compared to that of the unperturbed enriched anode biofilm (72.2 Ω). Confocal laser scanning microscopy observations showed that the shear-stress-enriched biofilms were entirely viable, whereas the unperturbed enriched anode biofilm consisted of a live outer layer covering a dead inner-core layer. Based on biomass and community analyses, the shear-stress-enriched biofilm had four times the biofilm density (136.0 vs. 27.50 μg DNA/cm^3^) and twice the relative abundance of *Geobacteraceae* (over 80 vs. 40%) in comparison with those of the unperturbed enriched anode biofilm. These results show that applying high shear stress during anode biofilm enrichment can result in an entirely viable and dense biofilm with a high relative abundance of exoelectrogens and, consequently, better performance.

## Introduction

Anodic microorganisms in microbial electrochemical systems (MESs) are biocatalysts that oxidize organic matter to transfer electrons to an electrode (Xiao et al., 2015; Hodgson et al., 2016). Normally, anodic microorganisms perform this process in the form of a biofilm. The anode biofilm is a complex aggregation of microbial communities and substances developed from planktonic microorganisms attached to the anode surface. It has been found that several factors can affect the formation, structure, and performance of anode biofilms, including the substrate concentration (Hari et al., 2017), electron acceptor (Ucar et al., 2017), anode solution (Liu et al., 2017), electrode potential (Bosire and Rosenbaum, 2017), and electric field intensity (Du et al., 2018).

The shear stress that arises from solution disturbance is an important factor affecting the formation, structure and performance of anode biofilms because of the physical force exerted on the anode biofilm and enhanced substance diffusion (substrate and metabolic end products) in the anode biofilm. Physical force can affect the attachment and detachment of microorganisms and the biofilm. For example, applying a high potential to the anode (Bosire and Rosenbaum, 2017), modifying the anode with positively charged compounds (Cheng and Logan, 2007) or improving the surface hydrophilicity (Du et al., 2017) can accelerate the attachment of bacteria due to the increased electrostatic attraction between anodic microorganisms and the electrode surface. Additionally, enhanced substance diffusion [either the substrate or metabolic end products (H^+^)] can increase the biomass, viability and performance of the anode biofilm. It has been shown that using a highly concentrated phosphate-buffered saline (PBS) solution as the anode electrolyte or increasing the pH of the anode electrolyte from a weak acid (pH = 6–7) to alkalinity (pH = 7–9) to mitigate proton accumulation in the anode biofilm or adjusting the gravity settling of planktonic bacteria and bioanode can increase the biomass, viability and current generation of anode biofilms (Liu et al., 2005; Cheng and Logan, 2007; Patil et al., 2011; Dhar et al., 2017; Li et al., 2017). In previous studies, we found that aerobically enriched anode biofilms with sufficient substance diffusion in the inner layer had a thicker inner layer and a higher current generation.

Nonetheless, few studies have examined the effect of shear stress on the formation, structure, and performance of anode biofilms. The impacts of shear stress on non-electrochemically active biofilms have been investigated. In general, a high shear stress usually results in thin, dense, and strong biofilms with low microbial diversity (Liu and Tay, 2002; Rickard et al., 2004; Celmer et al., 2008; Rochex et al., 2008). However, compared to these biofilms, the anode biofilm has substantially different characteristics, such as using an insoluble electron acceptor (mostly soluble molecules such as dissolved oxygen, nitrate and fermentation products are used for other biofilms) and the ability of long-distance extracellular electron transfer (EET) (Lovley, 2012). Therefore, these results are not directly translatable to the impacts of shear stress on anode biofilms.

In this study, we investigated the impacts of shear stress caused by nitrogen sparging (0–80 mL/min) on anode biofilm structure and performance. Solution disturbance caused by nitrogen sparging has been validated as an effective method to regulate shear stress (Celmer et al., 2008; Shen et al., 2013). For a fixed nitrogen sparging rate, we evaluated the anode performance in terms of startup time and linear sweep voltammetry (LSV). We then revealed the anode structure and characteristics with electrochemical impedance spectroscopy (EIS), confocal laser scanning microscopy (CLSM) and high-throughput 16S rRNA gene sequencing.

## Materials and Methods

### Configuration of Microbial Fuel Cells (MFCs)

Cubic dual-chamber air-cathode MFCs with a cation exchange membrane (CEM) were used in all experiments (Supplementary Figure S1). The MFC reactors were sealed with silica gel to prevent oxygen from leaking into the anode chamber. The anode and cathode were placed 6 cm apart at opposite ends of the MFC, and the CEM was placed at a distance of 2 cm from the cathode, resulting in anode and cathode working volumes of 30 and 14 mL. The anodes were disk-shaped graphite plates (surface area of 3.14 cm^2^, Shanghai Tansu, Co., Ltd., Shanghai, China) that were polished with 400-grit and 800-grit abrasive papers and 0.05 μm alumina oxide powder, then washed with 70% ethanol under ultrasonication and followed with deionized water. The nickel foam cathode contained an activated carbon catalyst, and a Poly tetra fluoroethylene (PTFE)-glass fiber diffusion layer was prepared as previously described (Cheng and Wu, 2013). The nitrogen sparging rates were controlled using gas flow meters (Kede Instruments, Co., Nanjing, China). The nitrogen flowed into the anode chamber from the bottom of the anode chamber and exited the chamber via a glass tube located at the top of the chamber. The opening for the gas was approximately 0.5 cm in front of the anode surface.

### MFC Inoculation and Operation

During startup and operation, nitrogen at a flow rate of 0, 10, 40, or 80 mL/min was continuously flowed through the anode chamber and the generated anode biofilms are referred to as A0, A10, A40, and A80, respectively. The gas outlet of A0 was continuously connected to an air pocket to balance the pressure. The air pocket and headspace of A0 was filled with high purity N_2_ and refreshed when replacing the anode solution. The source of inoculum was a 5-L MFC reactor inoculated with the primary clarifier overflow of a local wastewater treatment plant and fed with 50 mM PBS (2.45 g/L NaH_2_PO_4_⋅H_2_O, 4.58 g/L Na_2_HPO_4_, 0.31 g/L NH_4_Cl, and 0.13 g/L KCl) containing 15 mM acetic acid (Yang and Cheng, 2018). The inoculum was the effluent of the 5-L MFC mixed with 1.5 g/L acetate, 12.5 mL/L trace minerals and 5 mL/L vitamins. The anode chamber was immediately sparged after adding the inoculum. To maintain a constant shear stress on the anode surface, the anode chamber was continuously nitrogen sparged (except during the electrochemical analysis). During startup and operation, the electrodes were always connected with a 1000 Ω external resistance. The cell and anode potentials were recorded using a digital data-collection instrument (34970A, Agilent Technologies, Co., United States). After all MFCs had a stabilized voltage output, the solution was switched to 50 mM PBS containing 1.5 g/L acetate, 12.5 mL/L trace minerals, and 5 mL/L vitamins (Cheng and Logan, 2007) and replaced every 2 days. The inoculum and PBS was pre-nitrogen aerated to remove the dissolved oxygen. To avoid the impacts of oxygen on anode biofilm formation, when replacing the anode solution, the gas outlet of all the MFCs was connected to air pockets filled with high purity N_2_. Then, the nitrogen sparging was temporarily stopped and the anode solution was replaced through the pipe of gas inlet. The anode chamber was immediately sparged and the air pockets of A10, A40, and A80 were removed after replacing the anode solution. The cathode solution was 50 mM PBS during both startup and operation. All tests were conducted in a 30°C temperature-controlled room (except as noted).

### Electrochemical Analysis

The electrochemical analysis was performed after all the MFCs had a stabilized voltage output. LSV and EIS were performed in a three-electrode system using an electrochemical analyzer (Bio-Logic, Claix, France). The anode, cathode and Ag/AgCl electrode (0.220 mV vs. SHE) were employed as the working, counter and reference electrodes, respectively. The Ag/AgCl reference electrode was placed in close proximity to the anode. LSV tests were performed three times in the potential range from -0.6 to -0.1 V (vs. Ag/AgCl) at a scan rate of 1 mV/s, and the data collected in the third cycle were used for the analysis. EIS tests were conducted at the circuit voltage under an external resistance of 1000 Ω over the frequency range of 10^5^–0.01 Hz with a 10 mV sinusoidal perturbation.

### Biofilm Viability Observations and Calculation

The viability of the anode biofilms was observed by CLSM (LSM 780, ZEISS, GER). Before the observations, the anodes were rinsed with 50 mM PBS twice, stained with fluorochrome (LIVE/DEAD BacLight Bacterial Viability Kit, Invitrogen, Carlsbad, CA, United States) and rinsed with 50 mM PBS twice to remove any spare fluorochrome. At least two places were observed for each sample. The three-dimensional biofilm structure (z-stack) was reconstructed and analyzed using ZEN 2010 software (ZEISS, GER). The biofilm viability was calculated using ImageJ software according to the methods described by Solé et al. (2001) and Puyen et al. (2012). In short, every CLSM image was separated into two images containing only the red or green pixels. The separated images were transformed to binary images (black/white) using an automatic threshold and collected the binary pixels using an analyze function provided by ImageJ. The ratio of live cells to total cells was calculated as the green thresholded voxels (live cells) of the total green and red thresholded voxels (live and dead cells).

### Biomass and Microbial Community Analysis

After the CLSM observation, the anode biofilm was scraped from the graphite plate with a scalpel and moved to a 2 mL centrifuge tube. DNA was extracted using a DNA isolation kit (PowerSoil, United States) and quantified using a Qubit Fluorometer (Thermo Fisher Scientific, United States). The amount of biofilm biomass was evaluated based on the total DNA content of the anode biofilm (Liebeskind and Dohmann, 1994). The biofilm density was evaluated as the biomass of the biofilm volume. For community analysis, DNA extraction, PCR amplification (paired primers in the V3–V4 variable regions used for PCR amplification were F: 5′-ACTCCTACGGGAGGCAGCAG-3′ and R: 5′-GGACTACHVGGGTWTCTAAT-3′), fragment purification, database construction, and sequencing using the MiSeq Illumina sequencing platform, as previously described (Sun et al., 2015), were performed. The sequences were clustered into operational taxonomic units (OTUs) using UPARSE software based on >97% similarity. A representative sequence of OTUs was analyzed using the nucleotide collection (nr/nt) of the National Center for Biotechnology Information^1^ with the MegaBLAST algorithm.

## Results

### Effects of Shear Stress Enrichment on Startup and Electrochemical Performance of Anode Biofilms

**FIGURE 1:**
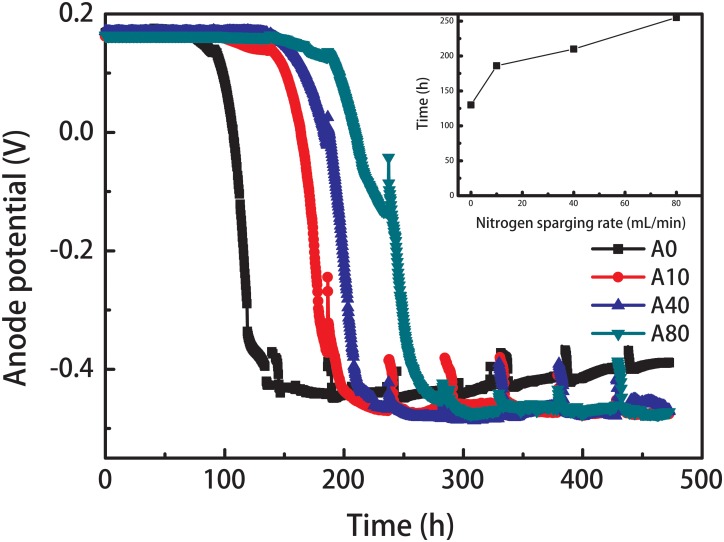
Anode potential of the anode biofilms enriched with different nitrogen sparging rates during startup. The inset shows the relationship between the nitrogen sparging rate and the time required for the anode potential reaching the stationary potential around –420 ± 20 mV.

The startup of all anodes showed a three-phase process (Figure 1): the lag phase (the anode potential only increased a few millivolts over several days), the rapid growth phase (the anode potential rapidly decreased to < -400 mV in approximately 2 days) and the stationary phase (the anode potential was maintained over -350 mV). The shear stress mainly extended the lag time, although the time of the rapid growth phase was also slightly increased by shear stress. The lag time of the anode with no nitrogen sparging (A0) was 100 h. Introducing 10–80 mL/min nitrogen sparging (A10, A40, and A80) in the anode chamber extended the lag phase to 150, 240, and 330 h, but the first maximum anode potential reaching the stationary phase increased slightly from -420 ± 10 mV (A0) to -440 ± 10 mV (A10, A40, and A80). Moreover, the stable potential of A0 gradually decreased from -420 ± 10 to -380 ± 10 mV with increasing operation cycles, while stable potentials of A10, A40, and A80 were maintained.

**FIGURE 2:**
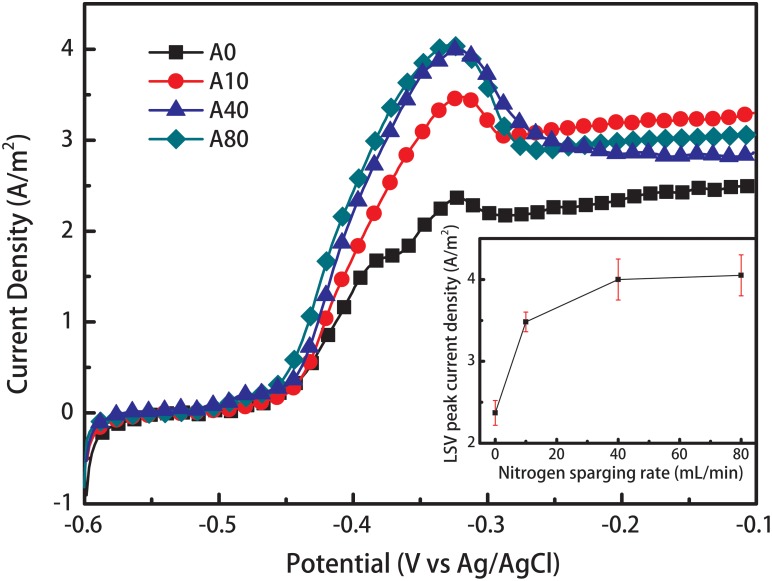
Linear sweep voltammetry (LSV) curves of the anodes enriched with different nitrogen sparging rates. The LSV tests was performed without nitrogen sparging, indicating that shear stress affected the anode biofilm structure and therefore influenced the anode performance. The inset shows the relationship between the nitrogen sparging rate and LSV peak current density.

The LSV curves of all anode biofilms showed a significant increase in current density starting at a potential of -0.48 V and reached a peak current at -0.32 V (Figure 2). The peak current density of A0 was 2.37 ± 0.15 A/m^2^. Introducing 10 and 40 mL/min nitrogen sparging into the anode chamber during anode biofilm formation increased the peak current density of the mature anode biofilm (A10 and A40) by 47 and 69% to 3.48 ± 0.12 and 4.0 ± 0.25 A/m^2^, respectively. Further increasing the nitrogen sparging rate to 80 mL/min during anode biofilm formation only slightly increased the peak current density of the mature anode biofilm (A80) to 4.05 ± 0.25 A/m^2^.

### Effects of Shear Stress Enrichment on Anode Resistance

**FIGURE 3:**
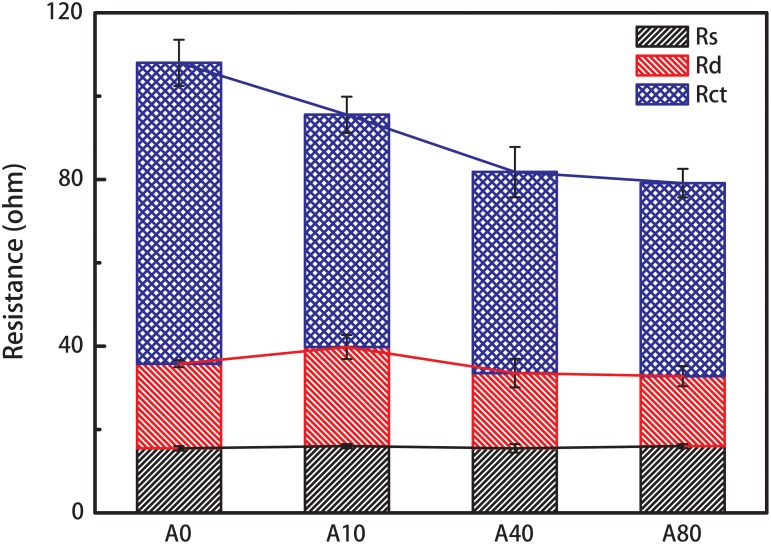
Anode resistance of the anodes enriched with different nitrogen sparging rates. Anode resistance was obtained from the Nyquist plots (Supplementary Figure S2) fitted using a two time-constant model, R_s_(R_ct_Q_1_)(R_d_Q_2_), in which R_s_ represents the anode resistance, R_ct_ represents the charge transfer resistance, R_d_ represents the diffusion resistance, and Q_1_ and Q_2_ are the constant phase angle elements.

The Nyquist plots (Supplementary Figure S2) obtained from the EIS test of the anodes in MFCs were usually fitted using a two time-constant model, R_s_(R_ct_Q_1_)(R_d_Q_2_), for the resistance estimation (Hou et al., 2014; Yang et al., 2017). In this model, R_s_ represents the anode resistance, R_ct_ represents the charge transfer resistance, R_d_ represents the diffusion resistance, and Q_1_ and Q_2_ are the constant phase angle elements. The shear stress enrichment mainly affected R_ct_, as R_s_ and R_d_ of all the anodes were approximately 16 ± 1 and 20 ± 4 Ω (Figure 3). The charge transfer resistance, R_ct_, of A0 was 72.2 ± 5.5 Ω and reduced by 23 and 33% to 55.8 ± 4.3 and 48.3 ± 6.0 Ω for the 10 and 40 mL/min nitrogen sparging-enriched anode biofilm (A10 and A40). R_ct_ continued to slightly reduce to 46.3 ± 3.4 Ω for the 80 mL/min nitrogen sparging-enriched anode biofilm (A80). The results of anode resistance were consistent with the LSV results.

### Effects of Shear Stress Enrichment on Biomass and Viability of Anode Biofilms

**FIGURE 4:**
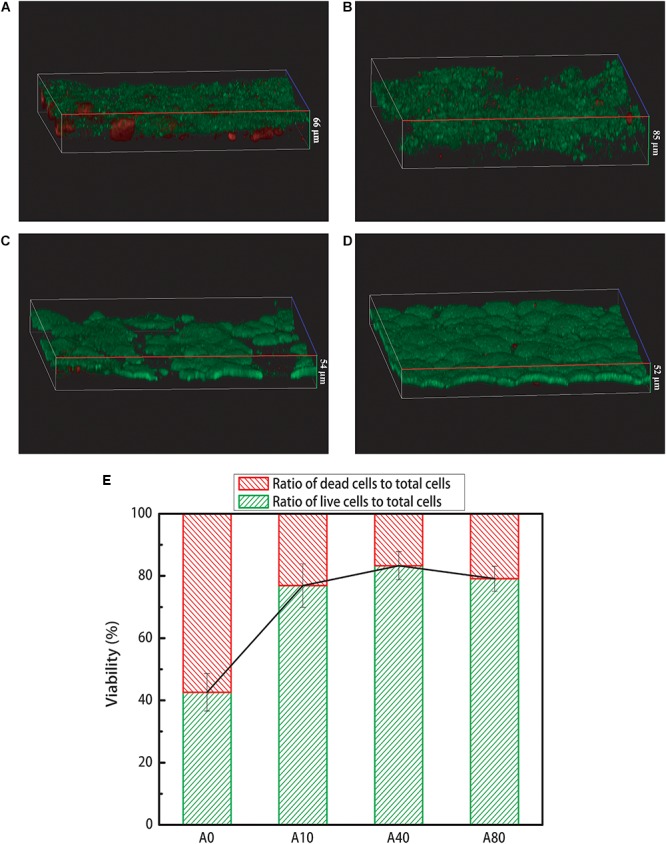
3D live/dead images of A0 **(A)**, A10 **(B)**, A40 **(C)**, and A80 **(D)** anode biofilms. **(E)** Ratio of live cells and dead cells to total cells based on pixel counting. The green color in **(A–D)** Represents live cells, whereas the red color represents dead cells. The numbers in **(A–D)** represent the thickness of anode biofilms.

Fluorescent staining was used to examine the viability of the biofilm, in which live cells are imaged in green and dead cells are imaged in red. The A0 anode biofilm showed a two-layer structure with a live outer layer on top of a dead inner-core layer (Figure 4A). Nitrogen sparging (10–80 mL/min) during anode biofilm formation and operation improved the biofilm viability of A10, A40 and A80, with one layer consisting mostly of live cells and only a small number of distributed dead cells (Figure 4B–D). The ratio of live cells to total cells of A0 was 42.6 ± 5% (Figure 4E). Introducing nitrogen sparging into the anode chamber during anode biofilm formation almost doubled the ratio of live cells to total cells of mature anode biofilm (A10, A40, and A80) to 76.9 ± 7, 83.3 ± 4.5, and 79.1 ± 4%. The biofilm thickness and morphology were also affected by nitrogen sparging. With an increasing nitrogen sparging rate, the thickness of the anode biofilms first increased from 66 μm (A0) to 85 μm (A10) and then decreased to 54 μm (A40) and 52 μm (A80). With no nitrogen sparging, the anode biofilm was flat. At a low nitrogen sparging rate of 10 mL/min, the anode biofilm appeared to be porous and rough. Upon further increasing the nitrogen sparging rate to 40 and 80 mL/min, the anode biofilms became flat again and appeared to have stronger aggregation. The total biomass of the anode biofilm was evaluated using its DNA contents. The biomass increased from 0.57 to 0.78, 1.56 and 2.22 μg with successive increases in nitrogen sparging rates from 0 to 10, 40, and 80 mL/min. Correspondingly, the cell densities increased from 27.50 to 29.22 and 92.0 and 136.0 μg DNA/cm^3^, respectively.

### Effects of Shear Stress Enrichment on Microbial Community of Anode Biofilms

**FIGURE 5:**
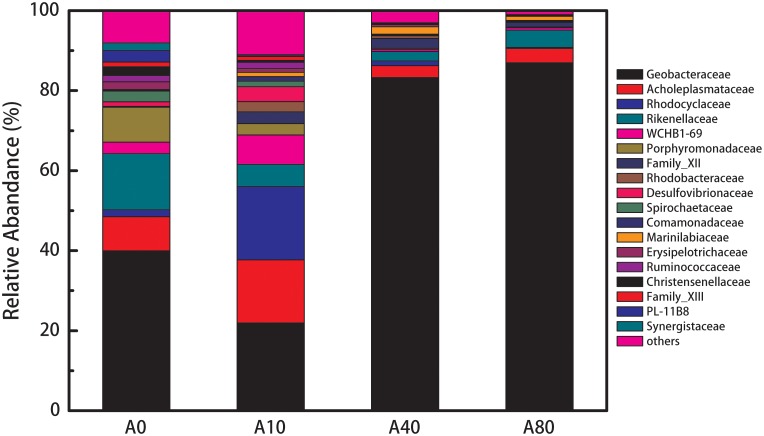
Composition and relative abundance of bacteria in A0-80 at the family level. The bacteria shown in the legend was selected based on a relative abundance higher than 0.5% of total population.

The A0 anode biofilm community mainly consisted of *Geobacteraceae* (40%), *Rikenellaceae* (14%), *Porphyromonadaceae* (8.8%), and *Acholeplasmataceae* (8.5%) (Figure 5). When 10 mL/min nitrogen sparging was introduced during the startup and operation, the relative abundance of *Geobacteraceae*, *Rikenellaceae*, and *Porphyromonadaceae* in the anode biofilm (A10) decreased to 22, 5.5, and 2.8%, respectively, and the relative abundance of *Rhodocyclaceae*, *Rhodobacteraceae*, *Desulfovibrionaceae*, *Comamonadaceae* increased from 1.7% to 18%, 0% to 2.6%, 1.1% to 3.7% and 0% to 1.1%, respectively (replication shown in Supplementary Figure S3). After further increasing the nitrogen sparging rate to 40 and 80 mL/min, the resulting anode biofilms (A40 and A80) were dominated by *Geobacteraceae* with relative abundances of 83 and 87%, respectively.

## Discussion

All MFCs were successfully started and operated under nitrogen sparging rates from 0 to 80 mL/min, indicating that exoelectrogens can form stable biofilms under shear stress. Interestingly, with an increase in the operation cycles, the stable potential of A0 gradually increased, but the stable voltages of A10, A40, and A80 remained constant (Figure 1). Even when the nitrogen sparging was stopped, A10, A40, and A80 had higher LSV peak current densities (Figure 2) than A0, indicating that shear stress affected the anode biofilm structure and therefore influenced the anode performance. The EIS results (Figure 3) showed that the increased anode performance of the MFCs with shear-stress-enriched anode biofilms was mainly due to their lower charge transfer resistances, indicating that the change in R_ct_ caused by the change in the anode biofilm structure is the key factor affecting anode performance. The observed anode biofilm structures that could affect R_ct_ were considered as three different factors: the viability structure, the physical structure and the microbial community structure.

With regard to the viability structure, the anode biofilms formed with nitrogen sparging (A10-80) mostly consisted of live cells in one layer (Figure 4B–D), though the A0 anode biofilm showed a two-layer structure with a live outer layer on top of a dead inner-core layer (Figure 4A). A two-layer structure generally leads to low anode performance. On the one hand, the live cells of the two-layer structure were less than that of the viable single layer with the same biomass. On the other hand, although the dead inner layer in the two-layer structure will not inhibit electron transfer from the live outer layer to the electrode, the electrochemical activity of the outer layer cells will be impaired by the dead inner layer, resulting in an increase in the charge transfer resistance (Sun et al., 2015, 2017; Dhar et al., 2017). The gradual decrease in the A0 stable potential was very possibly caused by the accumulation of dead cells in the inner layer of the anode biofilm. A similar decrease in current generation caused by the accumulation of dead cells in the inner layer of an anode biofilm was reported by Sun et al. (2015).

Regarding the physical structure, CLSM observations and biomass analyses showed that the biomass and biofilm density increased with the increasing nitrogen sparging rate. The EET of exoelectrogens may occur through conduction- or diffusion-based (mediated) mechanisms or a combination of mechanisms (Lovley, 2012). Regardless of the specific form of EET, increasing biomass and biofilm density will lead to reduced electrical resistance either by shrinking the spacing and increasing the electron shuttle density or by increasing the density and contact of components that exhibit metal-like conduction (Strycharz-Glaven et al., 2011). However, for nitrogen sparging rates over 40 mL/min, the biofilm density increased with the increasing nitrogen sparging rate, whereas the anode performance did not. This result might be because the charge transfer resistance R_ct_ includes the electron transfer resistance between microorganisms and microorganisms and the anode (Malvankar et al., 2012). Increasing the biofilm density will decrease the electron transfer resistance between microorganisms but minimally contribute to the electron transfer resistance between microorganisms and the anode. Pham et al. (2008) also reported that increasing the shear force from 10 to 120 s^-1^ resulted in two times more biomass and, therefore, the anode performance increased.

For the microbial community structure, *Geobacteraceae* dominated the anode biofilms on all anodes, especially the A40 and A80 anode biofilms (Figure 5). *Geobacteraceae* are known for their excellent electricity generation and long-range EET (Bond and Lovley, 2003) and are the dominant species in the anode biofilms of bioelectrochemical systems fed with acetate (Yates et al., 2012). The dominant presence of *Geobacteraceae* (over 80%) in the A40 and A80 compared to A0 (40%) contributed to the increased anode performance of A40 and A80. This increase in the *Geobacteraceae* percentage was believed to be altered by shear stress rather than oxygen. Introducing oxygen into the anode chamber during the operation of all the MFCs was strictly avoided. In the microbial community of A0, mostly of the dominating species, such as *Geobacteraceae* (40%) (Bond and Lovley, 2003), *Rikenellaceae* (14%) (Su et al., 2014), *Porphyromonadaceae* (8.8%) (Rosenberg et al., 2014), *Acholeplasmataceae* (8.5%) (Rosenberg et al., 2014) and Spirochaetaceae (2.7%) (Freundt et al., 1984), all were considered anaerobic. Notably, A0 had a higher abundance of *Geobacteraceae* than A10 (40 vs. 22%). However, compared to those in A0, many of the dominant families in the A10 anode biofilm have previously been reported to be exoelectrogens, such as *Rhodocyclaceae*, *Rhodobacteraceae*, *Desulfovibrionaceae*, and *Comamonadaceae*. *Rhodocyclaceae* species were found to be dominant in acetate-fed MFCs (Borole et al., 2009; Jangir et al., 2016), and some strains were confirmed to be exoelectrogens (Jangir et al., 2016). *Rhodobacteraceae* consists of chemoheterotrophs and photoheterotrophs, which are typical exoelectrogens (Kiely et al., 2011; Wong et al., 2016). *Desulfovibrionaceae* and *Comamonadaceae* have been widely found in MFCs, and their electricity generation ability has been confirmed (Xing et al., 2010; Eaktasang et al., 2016). In contrast, of the dominant families in A0, members of *Rikenellaceae* are anaerobic fermentation bacteria that tend to use complicated substrates such as peptone, yeast extract, maltose and glucose, but cannot exploit some simple organic matter such as formic acid, acetate, and ethyl alcohol (Su et al., 2014). Thus, as the provided substrate was acetate, the high relative abundance of *Rikenellaceae* might be related to the accumulation of dead cells. The next dominant family, *Porphyromonadacea*, was found in abundance in the anode biofilm of dual-chamber MFCs (Sotres et al., 2015), but the electricity generation ability of this family has not been confirmed. *Acholeplasmataceae* has not been reported in MFCs. In summary, the A10 biofilm possibly had a higher abundance of exoelectrogens than A0, resulting in the higher anode performance.

In this study, we showed that increasing shear stress could increase anode performance. Increasing the shear stress requires additional energy consumption, and optimization of the flow rate and power generation is needed. Moreover, we revealed, for the first time, that shear stress helps maintain a viable anode biofilm. The viability of the anode biofilm was found to be altered by the stable current density of the anode biofilm or the PBS concentration of the anode solution (Sun et al., 2015, 2017; Dhar et al., 2017). When the stable current density was lower than 2.3 A/m^2^ or the PBS concentration was lower than 10 mM, the anode biofilm formed a two-layer structure with a live outer layer covering a dead inner layer. In contrast, when the stable current density of the anode biofilm was higher than 4.8 A/m^2^ or the PBS concentration was higher than 100 mM, the anode biofilm was mostly a viable single layer. Thus, to maintain a high-performance and viable anode biofilm, an anode biofilm should be operated with a high current density or high PBS concentration. However, in application, especially for wastewater treatment, the highly variable organic matter contents and low concentrations of organic matter and ionic strengths usually result in little current production, impacting the long-term viability of the anode biofilm. The one-layer viable anode biofilm structure under nitrogen sparging provides an instructive method for maintaining a high-viability active anode biofilm at low current density (1.1 ± 0.1 A/m^2^ in this work) and low PBS concentration (50 mM PBS in this work), facilitating high performance and sufficient COD removal in MESs for applications.

## Conclusion

Anodes were started and operated under nitrogen sparging rates from 0 to 80 mL/min. Increasing the nitrogen sparging rate from 0 to 10, 40, and 80 mL/min improved the anode performance by 47, 69, and 69%, respectively (2.37 ± 0.15 vs. 3.48 ± 0.12, 4.0 ± 0.25 and 4.05 ± 0.25 A/m^2^). The improved anode performance was attributed to the viability, physical and microbial community structures of the anode biofilms. For the viability structure, the unperturbed enriched anode biofilm showed a two-layer structure with a live outer layer on top of a dead inner-core layer. The perturbed enriched biofilms exhibited only a single viable layer. Regarding the physical structure, the biomass and biofilm density increased with the increasing nitrogen sparging rate. For the microbial community structure, compared to that of the unperturbed enriched anode biofilm, the low nitrogen sparging rate (10 mL/min)-enriched anode biofilm had a higher abundance of exoelectrogens, such as *Rhodocyclaceae*, *Rhodobacteraceae*, *Desulfovibrionaceae*, and *Comamonadaceae*, and the high nitrogen sparging rate (40 and 80 mL/min)-enriched anode biofilms had a higher abundance of *Geobacteraceae* (over 80 vs. 40%).

## Author Contributions

JY and SC: study design and article writing. JY, CL, YS, and HH: experiments. JY, SC, CL, YS, and HH: data analysis.

## Conflict of Interest Statement

The authors declare that the research was conducted in the absence of any commercial or financial relationships that could be construed as a potential conflict of interest.

## Footnotes

1 www.ncbi.nlm.nih.gov/BLAST/
