# Supplementary material for: Shear Stress Affects Biofilm Structure and Consequently Current Generation of Bioanode in Microbial Electrochemical Systems (MESs)
Source: Front Microbiol. 2019 Mar 6;10:398. doi: 10.3389/fmicb.2019.00398 (PMC6415583; doi:10.3389/fmicb.2019.00398)
Supplement: Supplementary file 1 [file Table_1.docx]

**Supplementary Information**

Submitted to: *FRONTIERS IN MICROBIOLOGY*

**Shear Stress Affects Biofilm Structure and Consequently Current Generation of Bioanode in Microbial Electrochemical Systems (MESs)**

***Jiawei Yang, Shaoan Cheng^*^, Chaochao Li, Yi Sun and Haobin Huang***

*State Key Laboratory of Clean Energy, Department of Energy Engineering, Zhejiang University, Hangzhou 310027, PR China*

* **Correspondence:**

Dr. Shaoan Cheng

E-mail: shaoancheng@zju.edu.cn;

**
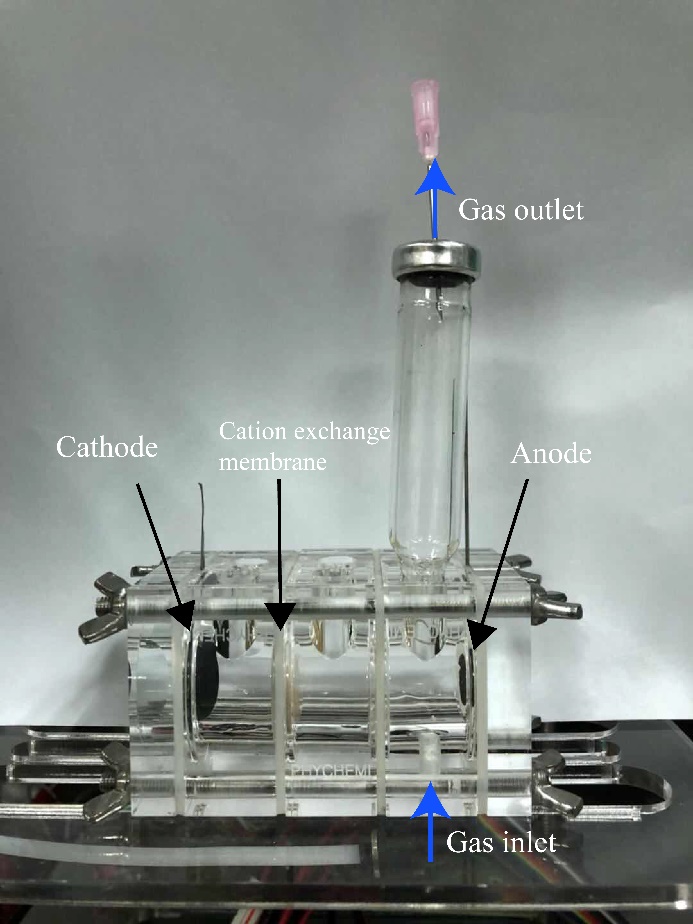
**

**FIGURE S1 Photograph of the MFC.**


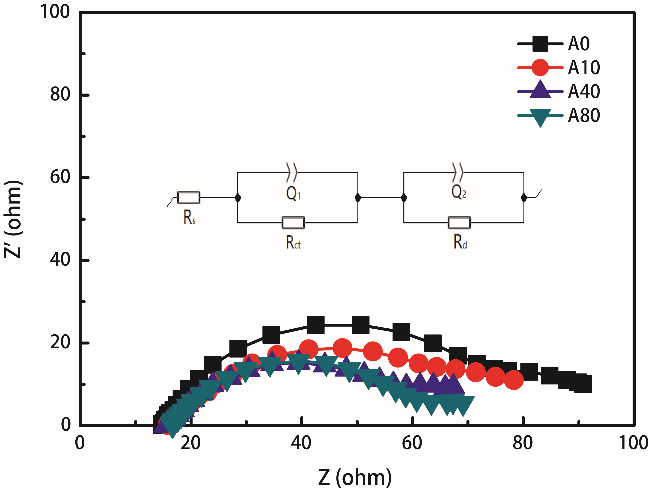


**FIGURE S2 Nyquist plots of the EIS spectra of the MFCs.** The inset shows the equivalent circuit that was used to estimate the anode resistance.


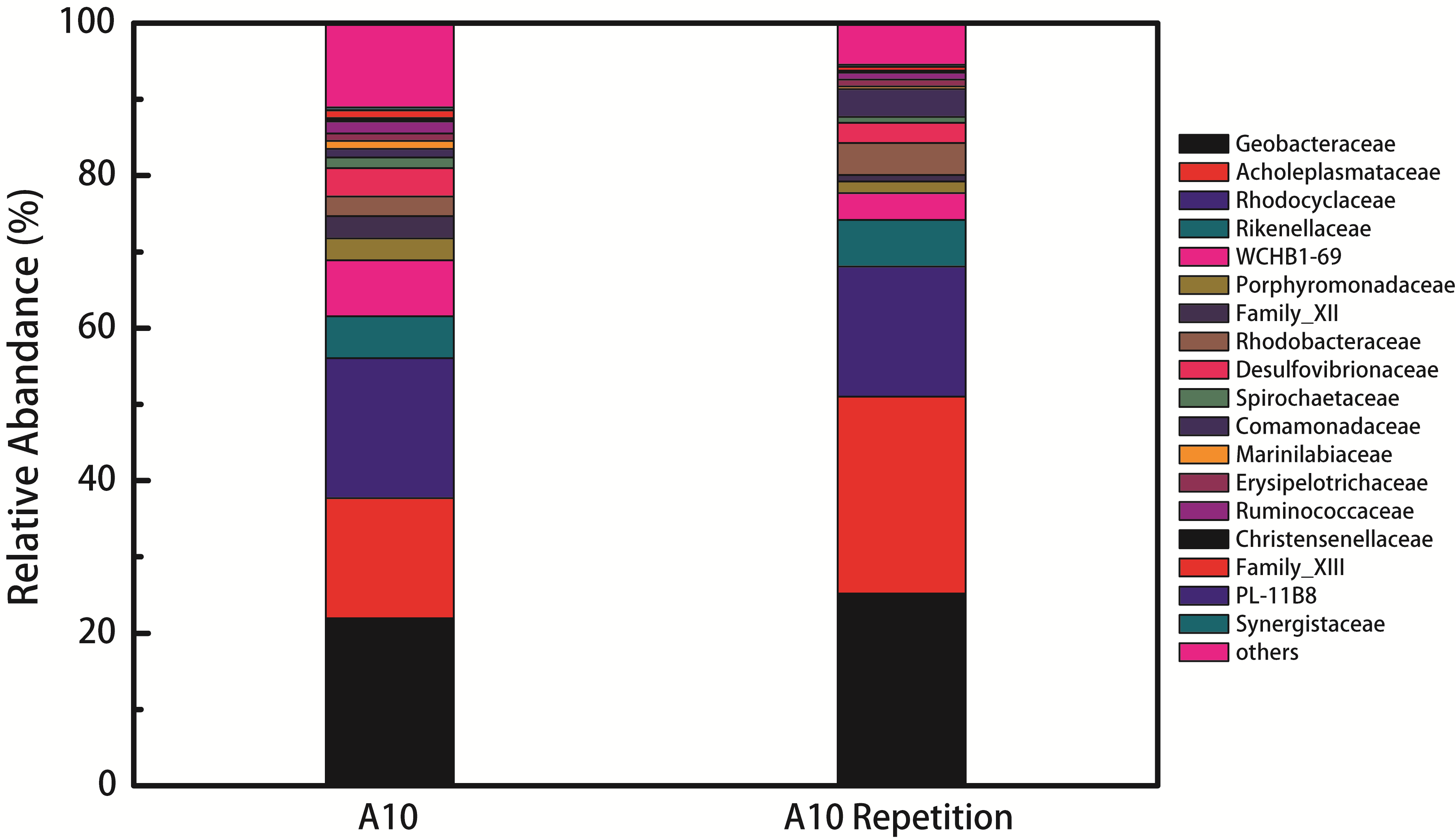


**FIGURE S3 Composition and relative abundance of bacteria in A10 at the family level.**
